# First report of a carbapenemase OXA-48-producing *Hafnia alvei* clinical isolate

**DOI:** 10.1099/acmi.0.000498.v3

**Published:** 2023-06-22

**Authors:** Laura Sevillano, Cristhian Herrera, Álvaro Valdes, Ángela de la Hoz, Laura Cardeñoso, Diego Domingo, Maria Auxiliadora Semiglia

**Affiliations:** ^1^​ Servicio de Microbiología, Hospital Universitario de La Princesa, Madrid, Spain; ^2^​ Servicio de Cirugía General y Digestivo, Hospital Universitario de La Princesa, C/Diego de León, 62. 28006, Madrid, Spain

**Keywords:** carbapenem, OXA-48, *Hafnia alvei*, carbapenemase

## Abstract

**Introduction.:**

Carbapenems are usually used in the treatment of infections caused by cephalosporin-resistant *Enterobacterales *; however, the increase in carbapenem-resistant *Enterobacterales* (CRE) has become one of the most important problems in public health. *Hafnia alvei* is associated with intestinal and extraintestinal infections, especially in patients with any chronic disease or some type of immunosupression. *H. alvei* is resistant to first-generation aminopenicillins and cephalosporins owing to the β-lactamase (Amp C) in their chromosome; the only carbapenem-resistant *Hafnia* strain described until now was due to a lack of the OmpK36 protein that plays an important role in permeability to carbapenems.

**Case presentation.:**

We present the case of a 65-year-old male diagnosed with acute lithiasic cholecystitis. Culture of the biliary prosthesis yielded a OXA-48-producing *H. alvei* that was identified by MALDI-TOF (matrix-assisted laser desorption/ionization-time of flight) MS. Carbapenemase production was detected by immunochromatography and confirmed by sequencing.

**Conclusion.:**

To our knowledge, this is the first report of OXA-48-producing *H. alvei* probably obtained by horizontal transfer from *Enterobacter cloacae* OXA-48 isolated in previous samples.

## Data Summary

No data were generated during this research.

## Introduction

The rise in consumption of carbapenems has promoted the emergence of carbapenem-resistant *Enterobacterales* (CRE) through the diffusion of plasmid-borne carbapenemases. This has become a major challenge for healthcare systems because carbapenems are the last line of defence against infections caused by third- and fourth-generation cephalosporin- resistant *Enterobacterales *. As a consequence, treatment options for CRE are alarmingly limited [1–3]. Among carbapenemases, OXA-48 is the most prevalent one used in Spain [4].

*Hafnia alvei* is a facultatively anaerobic rod-shaped Gram-negative bacterium that belongs to the family *Hafniaceae* in the order *Enterobacterales* [5]. It is widely distributed in nature and is part of the intestinal commensal microbiota of many animals and considered an opportunistic pathogen. In humans, *H. alvei* is a normal inhabitant of the intestinal tract, although some researchers also consider it a commensal of the respiratory tract [5]. It has been shown to be predominantly associated with several intestinal disorders, including gastroenteritis and also extraintestinal disorders (bacteraemia, pneumonia) [6–9]. Its role in infectious pathology is becoming more frequent due to better identification in microbiology laboratories [10–13].

*H. alvei* has an inducible chromosomal β-lactamase (Amp C) with cephalosporinase activity, which generally confers resistance to first-generation aminopenicillins and cephalosporins, but not to third- or fourth-generation cephalosporins. In 2010 a strain of *Hafnia* resistant to carbapenems was isolated. This resistance was due to the lack of a major outer-membrane protein, OmpK36, that plays a role in permeability to carbapenems in *Enterobacterales* [14].

In this study we report a clinical case of a 65-year-old male with an intra-abdominal infection in which the first strain of *Hafnia alvei* harbouring OXA-48 carbapenemase was isolated.

## Case Presentation

We report a case of a 65-year-old male with a medical history of hepatitis C (Genotype 1b, F0-1) infection treated with Exviera plus Viekirax (DAA) with a sustained virological response (SVR) and porphyria cutanea tarda also successfully treated.

The patient was admitted to the hospital with a 72 h history of abdominal pain in the right upper quadrant, exacerbated by food intake and partially relieved by vomiting; other remarkable symptoms such as acholia, choluria and a body temperature of 38 °C were documented. On inspection, the patient presented skin and conjunctival jaundice, and abdominal examination revealed diffuse pain upon palpation predominantly in the right upper quadrant with positive Murphy’s sign.

**Fig. 1.**
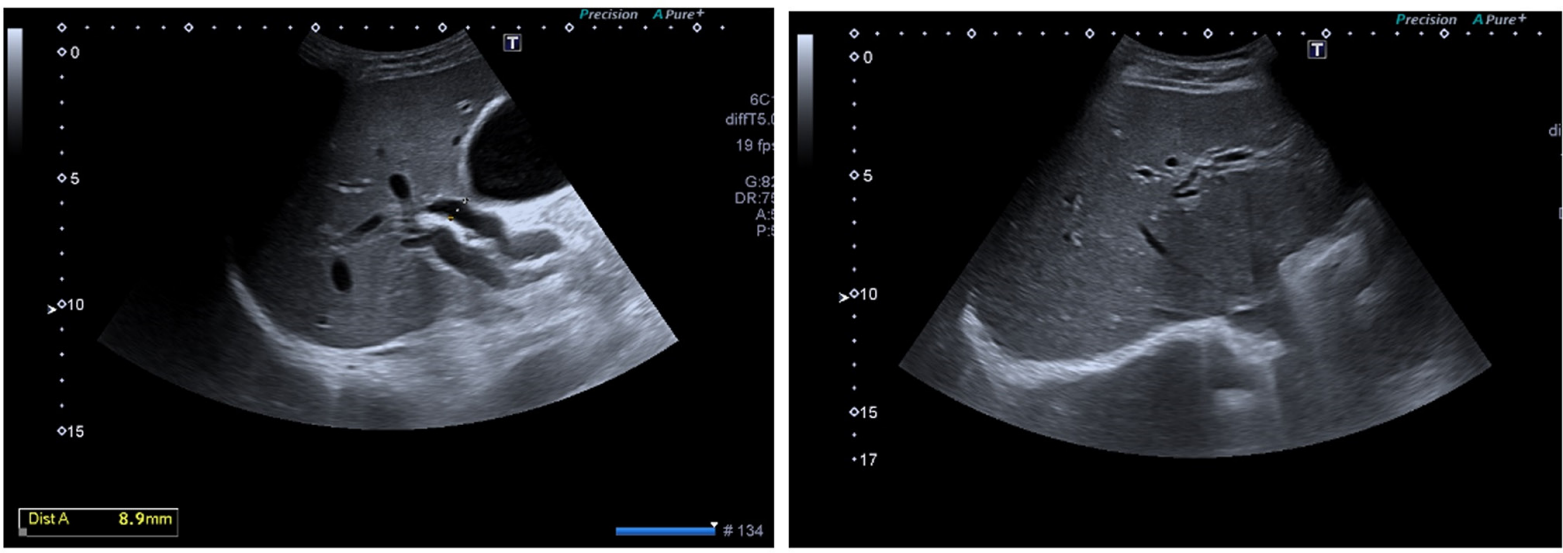
Abdominal ultrasound: acute cholecystitis and intra- and extra-hepatic biliary dilatation.

Blood analysis data on admission were: leukocytes 6.72×10^3^ mm^−3^ (neutrophils 83.4%), platelets 184×10^3^ mm^−3^, total bilirubin 6.17 mg dl^−1^, gamma-glutamyl transferase (GGT) 289 U l^–1^, glutamic oxaloacetic transaminase (GOT)/aspartate transaminase (AST) 148 U l^–1^ and glutamate pyruvate transaminase (GPT)/alanine transaminase (ALT) 439 U l^–1^. An ultrasound scan of the patient’s abdomen (Fig. 1) indicated an acute lithiasic cholecystitis and an intra- and extrahepatic dilatation of bile duct, without the identification of an obstructive cause.

**Fig. 2.**
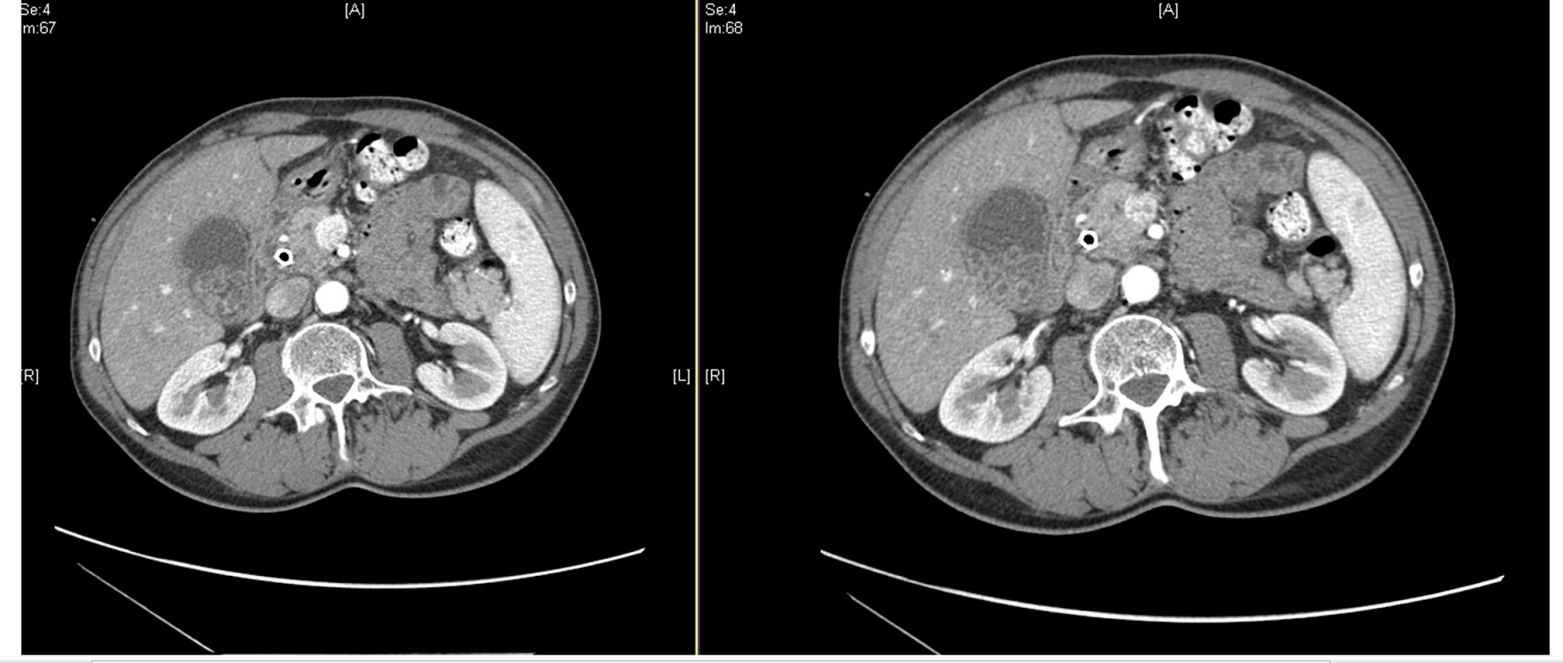
Abdominal CT scan: multiple cholelithiasis with acute cholecystitis.

Given the patient’s symptoms and the results of complementary tests, the patient was empirically treated with piperacillin/tazobactam and was admitted to the hospital. During hospitalization, an endoscopic retrograde cholangiopancreatography (ERCP) showed a stenosis at about 20 cm from the distal common bile duct, and a biliary endoprosthesis was inserted. An abdominal computed tomography scanner (Fig. 2) revealed a lithiasic gallbladder without lesions suspicious of malignancy. The patient was discharged for detailed medical studies but required hospitalization 3 weeks later in the context of acute cholecystitis suspicion. Percutaneous cholecystectomy was performed and piperacillin/tazobactam treatment was initiated (4 g every 8 h for 14 days). Three biliary samples were obtained for microbiological studies. Seven days later, the patient was submitted to a pancreatoduodenectomy and the prosthesis previously implanted was also sent for microbiological procedures.

Cultures were performed following conventional procedures in the media and environment recommended. Identification and susceptibility tests were performed using matrix-assisted laser desorption/ionization-time of flight (MALDI-TOF) MS (Bruker Daltonics) and MicrosCan Walkay panels (Beckman Coulter), respectively. MICs were interpreted according to EUCAST 2020 criteria [15], and carbapenemase production was detected by immunochromatography (RESIST-4 OKNV carbapenemase; Coris BioConcept).

The three bile samples yielded the culture of OXA-48 carbapenemase-producing *Enterobacter cloacae* and in two of them *Enterococcus faecium* was also isolated. OXA-48 carbapanemase-producing *Klebsiella pneumoniae* and *H. alvei* were obtained from the culture of the biliary prosthesis, as well as *Enterococcus faecium* (Table 1 gives a summary of microbiological results of the different samples). After microbiology results, antibiotic treatment was changed to amikacin (300 mg every 8 h) and meropenem (1 g every 8 h), for 6 days. Results of the antibiogram (Table 2) showed carbapenem resistance that revealed the presence of a carbapenemase. Carbapenemase production was confirmed by immunochromatography (RESIST-4 OKNV carbapenemase; Coris BioConcept) being positive for OXA-48 carbapenemase. The *H. alvei* strain was sent to the National Center for Clinical Microbiology (Carlos III Research Institute, Madrid) that confirmed OXA-48 production by sequencing. Antimicrobial treatment was changed to ceftazidime/avibactam (2 g every 8 h for 10 days) and tygecicline (50 mg every 12 h for 5 days) due to an increase in procalcitonin and C-reactive protein (CRP); both parameters decreased after administration of the new treatment. Eighteen days after the pancreatoduodenectomy, the patient was discharged and attended at outpatient consultation area.

**Table 1.** Microorganisms isolated in the different samples

Date	Sample	Isolate 1	Isolate 2	Isolate 3
03.01.2021	Bile	* E. cloacae* OXA-48		
04.01.2021	Bile	* E. cloacae* OXA-48	* E. faecium *	
12.01.2021	Bile	* E. cloacae* OXA-48	* E. faecium *	
21.01.2021	Biliary prosthesis	* K. pneumoniae* OXA-48	*H. alvei O*XA-48	* E. faecium *

**Table 2.** MIC distribution (mg l^–1^)

	AMP	XL	PTZ	ETP	MP	IMP	GM	TO	SXT	CIP	CZ/AV
* E. cloacae* OXA-48	>8	>8/4	>16	>1	2	2	>4	>4	>4/76	>1	2
* K. pneumoniae* OXA-48	>8	>8/4		>1	2	2	<2	<2	<2/38	<0.25	0.19
* H. alvei* OXA-48	>8	>8/4		>1	2	<1	<2	<2	<2/38	<0.25	

AMP, Ampicillin; CAZ, ceftazidime; CIP, ciprofloxacin; CTX, cefotaxime; CZ/AV, ceftazidime/avibactam; ETP, ertapenem; GM, gentamicin; IMP, imipenem; MP, meropenem; PTZ, piperacillin/tazobactam; SXT, cotrimoxazole; TO, tobramycin; XL, amoxicillin/clavulanic.

## Discussion

In this paper we describe the first isolation of an *H. alvei* clinical isolate harbouring a OXA-48-like carbapenemase, and the repercussions linked to this finding. The clinical significance of *H. alvei* has been in question since its discovery [16] although its role in clinical diseases should be considered as with any *Enterobacterales *. In recent years, infections due to this microorganism have increased mainly in infections with an intra-abdominal focus and in patients with some type of immunosuppression such as transplant patients [10, 16, 17].

Many studies have indicated that *H. alvei* is usually resistant to ampicillin, penicillin, piperacillin, first- and second-generation cephalosporins, and amoxicillin-clavulanic and piperacillin-tazobactam combinations, due to the Amp C betalactamase. The vast majority of studies show that *H. alvei* is susceptible to aminoglycosides, quinolones, carbapenems, third-generation cephalosporins and trimethoprim-sulfamethoxazole combinations. Regarding colistin, many studies have indicated that *H. alvei* is naturally resistant to colistin, like other genera of *Enterobacterales* such as *Proteus *, *Providencia *, *Morganella* and *Serratia* [5].

Today, antibiotic resistance is the most important problem in public health. Carbapenem resistance is of particular importance because carbapenems have the widest spectrum of activity and are the agents of choice against multidrug-resistant (MDR) Gram-negative pathogens [18, 19]. There are at least three major mechanisms of carbapenem resistance in *Enterobacterales *: enzyme production (carbapenemase), production of efflux pumps and porin mutations [20, 21]. Of these, carbapenemase production is the main resistance mechanism; there are three main groups of carbapenemases: KPC (*Klebsiella pneuominae* carbapenemase), MBLs (metalo-β-lactamases) and OXA-48-like [20]. There is geographical diversity in the distribution of these carbapenemases, OXA-48 being the most common in Europe. The three groups of carbapenemases are plasmid-mediated, indicating easy horizontal transference and a faster dispersion of carbapenem resistance [20].

Acquisition of blaOXA-48-like genes has been identified only in *Enterobacterales *, but not in other Gram-negative bacteria such as *A. baumannii* or *P. aeruginosa*, even though other carbapenem-hydrolysing class D β-lactamase (CHDL)-encoding genes are identified in those species. The blaOXA-48 gene is located on ~70 kb plasmids that are self-transferable and do not carry additional resistance determinants [22].

Initially, OXA-48 was identified in a carbapenem-resistant *Klebsiella pneumoniae* isolate from Turkey in 2001, and outbreaks of OXA-48-producing *Enterobacterales* have since been reported worldwide. Today, OXA-48 is one of the most prevalent carbapenemases worldwide. The global dissemination of OXA-48-producing MDR gram-negative rods and the presence of mobile genetic elements in a broad range of species have established OXA-48 as a major public health threat in recent years [23].

Different studies have provided strong evidence for both within-host interspecies and between-host dissemination of plasmid-based OXA-48 during different nosocomial outbreaks [23, 24]. In the case described here, the first OXA-48-producing isolate was *E. cloacae* in the first bile sample cultured; 3 weeks later, OXA-48-producing *H. alvei* and *K. pneuomoniae* were identified, resembling a case of within-host interspecies dissemination of the OXA-48 plasmid. The acquisition of carbapenemases as OXA-48 in new species such as *H. alvei *, as described in this paper, may be a determinant in the propagation of the enzyme and play an important role in the treatment of these infections.

These findings support the importance of looking for new antibiotics to fight against MDR microorganisms in the treatment of infections.

There are different treatment options to treat infections caused by these MDR bacteria, and these options include the repurposing of existing antibiotics, dual therapies, and the development of new antibiotics and new β-lactamase inhibitors [18–20]. A major problem with OXA-48 is that most inhibitors do not act against it [20, 25] and they have a high mutation rate. In the last few years, new agents with activity against carbapenem-resistant bacteria have been approved for clinical use by the Food and Drug Administration and European Medicines Agency, including plazomicin, eravacycline, cefiderocol, temocillin, ceftazidime-avibactam, ceftolozane-tazobactam, meropenem-vaborbactam and imipenem-relebactam. The use and activity spectrum of these new antibiotics have been summarized in different reviews [18–21, 25, 26].

In conclusion, in this paper we describe the first clinical isolate of an OXA-48-producing *H. alvei *, probably obtained by horizontal transfer from other species of the same order, in the context of an abdominal infection in which the high bacterial inoculum can promote plasmidic dissemination of the enzyme.
